# Learning curves in robotic and laparoscopic colorectal surgery: a systematic review

**DOI:** 10.1007/s11701-026-04024-x

**Published:** 2026-10-03

**Authors:** Fabio Nocera, Felicitas Wiedemann, Philip C. Müller, Adrian T. Billeter, Fiorenzo V. Angehrn, Beat P. Müller, Daniel C. Steinemann, Christoph Kuemmerli

**Affiliations:** 1 Department of Surgery, Clarunis - University Digestive Health Care Center Basel - Clarunis, Site St. Claraspital, Kleinriehenstrasse 30, Basel, 4058 Switzerland; 2 Medical Faculty, University of Basel, Basel, Switzerland

**Keywords:** Robotic surgery, Colorectal surgery, Learning curve, Laparoscopic surgery, Surgical training, CUSUM

## Abstract

**Objective:**

To evaluate and compare learning-curve assessment in laparoscopic and robotic colorectal surgery and to develop a standardized framework for future learning-curve reporting.

**Background:**

Learning curves in minimally invasive colorectal surgery are widely reported, but inconsistent definitions limit interpretability and comparability. This systematic review analyzed learning-curve assessment in laparoscopic and robotic colorectal surgery and proposes a standardized framework.

**Methods:**

MEDLINE, Web of Science, and CENTRAL were systematically searched according to PRISMA guidelines (registered in PROSPERO, CRD42026128860). Included were randomized, prospective, and retrospective studies on learning curves in colorectal surgery. Primary outcome was the number of procedures required to reach the initial learning phase. Secondary outcomes included definitions of learning curves, analytical methods, and classification of learning phases. Analyses were stratified by procedure type (colon vs. rectal resections), and pooled breakpoint regression was used to identify learning phases.

**Results:**

A total of 114 studies comprising 167 learning-curve analyses were included. CUSUM or risk-adjusted CUSUM was used in 66.4% of analyses, whereas 33.3% applied arbitrary definitions; operative time was the most frequently used endpoint (79.6%). Operative-time plateaus were identified after 38 colonic and 56 rectal procedures. Reported median competency thresholds were numerically lower for robotic than laparoscopic surgery in colonic resections (24 vs. 30 cases; *p =* 0.659) and rectal resections (28 vs. 38 cases; *p =* 0.140), but these differences were not statistically significant.

**Conclusions:**

Learning-curve assessment in minimally invasive colorectal surgery remains heterogeneous. Robotic series report numerically lower competency thresholds than laparoscopic series; however, this likely reflects skill transfer from prior laparoscopic experience and modern training pathways rather than an intrinsically shorter learning curve of the robotic platform. Differences in prior surgeon experience, training, and study era limit direct comparison. Standardized assessment and prospective studies with comparable baseline exposure are needed.

**Supplementary Information:**

The online version contains supplementary material available at https://doi.org/10.1007/s11701-026-04024-x.

## Plain Language

Learning curves in minimally invasive colorectal surgery are widely reported, but inconsistent definitions limit interpretability and comparability. This systematic review analyzed learning-curve assessment in laparoscopic and robotic colorectal surgery and proposes a standardized framework. MEDLINE, Web of Science, and CENTRAL were systematically searched according to PRISMA guidelines (registered in PROSPERO, CRD42026128860). Included were randomized, prospective, and retrospective studies on learning curves in colorectal surgery. Primary outcome was the number of procedures required to reach the initial learning phase. Secondary outcomes included definitions of learning curves, analytical methods, and classification of learning phases. Analyses were stratified by procedure type (colon vs. rectal resections), and pooled breakpoint regression was used to identify learning phases. A total of 114 studies comprising 167 learning-curve analyses were included. CUSUM or risk-adjusted CUSUM was used in 66.4% of analyses, whereas 33.3% applied arbitrary definitions; operative time was the most frequently used endpoint (79.6%). Operative-time plateaus were identified after 38 colonic and 56 rectal procedures. Reported median competency thresholds were numerically lower for robotic than laparoscopic surgery in colonic resections (24 vs. 30 cases; p = 0.659) and rectal resections (28 vs. 38 cases; p = 0.140), but these differences were not statistically significant. Learning-curve assessment in minimally invasive colorectal surgery remains heterogeneous. Robotic series report numerically lower competency thresholds than laparoscopic series; however, this likely reflects skill transfer from prior laparoscopic experience and modern training pathways rather than an intrinsically shorter learning curve of the robotic platform. Differences in prior surgeon experience, training, and study era limit direct comparison. Standardized assessment and prospective studies with comparable baseline exposure are needed.

**Supplementary Information:**

The online version contains supplementary material available at https://doi.org/10.1007/s11701-026-04024-x.

## Introduction

Minimally invasive surgery has become an established approach in colorectal surgery, with laparoscopic techniques demonstrating oncological safety and favorable perioperative outcomes compared with open surgery [1–3]. More recently, robotic platforms have been increasingly adopted, particularly for technically demanding colorectal procedures. The ROLARR trial demonstrated comparable surgical and short-term oncological outcomes between robotic and laparoscopic rectal surgery [4], while other studies have reported favorable pathological and perioperative outcomes associated with robotic surgery [5].

The adoption of any new surgical technique is accompanied by a learning process during which operative efficiency and clinical outcomes may improve with increasing experience. Although robotic surgery is frequently perceived to have a shorter learning curve than conventional laparoscopy, the available evidence is difficult to interpret because learning curves are defined and assessed inconsistently across studies [6, 7]. In addition, direct comparisons between laparoscopic and robotic learning curves are confounded by differences in baseline surgeon experience and study era. Early laparoscopic series often reflect de novo acquisition of minimally invasive skills, whereas robotic adoption frequently occurs among surgeons who are already proficient in laparoscopic surgery [6, 8, 9].

Despite increasing interest in learning-curve assessment in colorectal surgery, no uniform definition or standardized method of assessment exists. Studies use different endpoints, statistical methods, and thresholds to define competency or proficiency, substantially limiting comparability and clinical interpretation [10–13]. A three-phase model distinguishing competency, proficiency, and mastery has been proposed in other surgical specialties [10, 11], but a comparable comprehensive framework for colorectal surgery is lacking.

The aim of this systematic review was therefore to characterize how learning curves are assessed in laparoscopic and robotic colorectal surgery, compare reported learning-curve thresholds and outcomes across approaches and procedure types, and propose a standardized framework for defining and reporting distinct learning phases.

## Methods

### Systematic literature search methodology

This systematic review adheres to the methodological standards outlined in the Cochrane Handbook for Systematic Reviews of Interventions and has been reported in accordance with the PRISMA guidelines [14, 15]. The review protocol was registered in the PROSPERO international prospective register of systematic reviews (PROSPERO-ID: CRD42026128860). Literature was systematically searched using the MEDLINE, Web of Science, and Cochrane Central Register of Controlled Trials (CENTRAL) databases [16]. Search terms were combined using Boolean operators and medical subject headings (MeSH). No language restrictions were applied during the literature search; however, studies without full-text availability in English were excluded. In addition, cross-referencing and manual screening of the bibliographies of eligible studies were performed to identify further relevant publications for inclusion in the review. A systematic literature search was performed in the MEDLINE database (via PubMed) on August 14, 2024, employing the following search strategy: (Colorectal Surgery OR Colonic Resection) AND (Open OR Laparoscopic OR Minimal-invasive OR Robotic Surgery OR Robotic-assisted Surgery OR DaVinci) AND (Learning Curve OR Proficiency OR Mastery OR Competency OR Learning Phase) NOT (Pancreatic Surgery OR Liver Surgery OR Animal).

### Study selection and data extraction

**Fig. 1:**
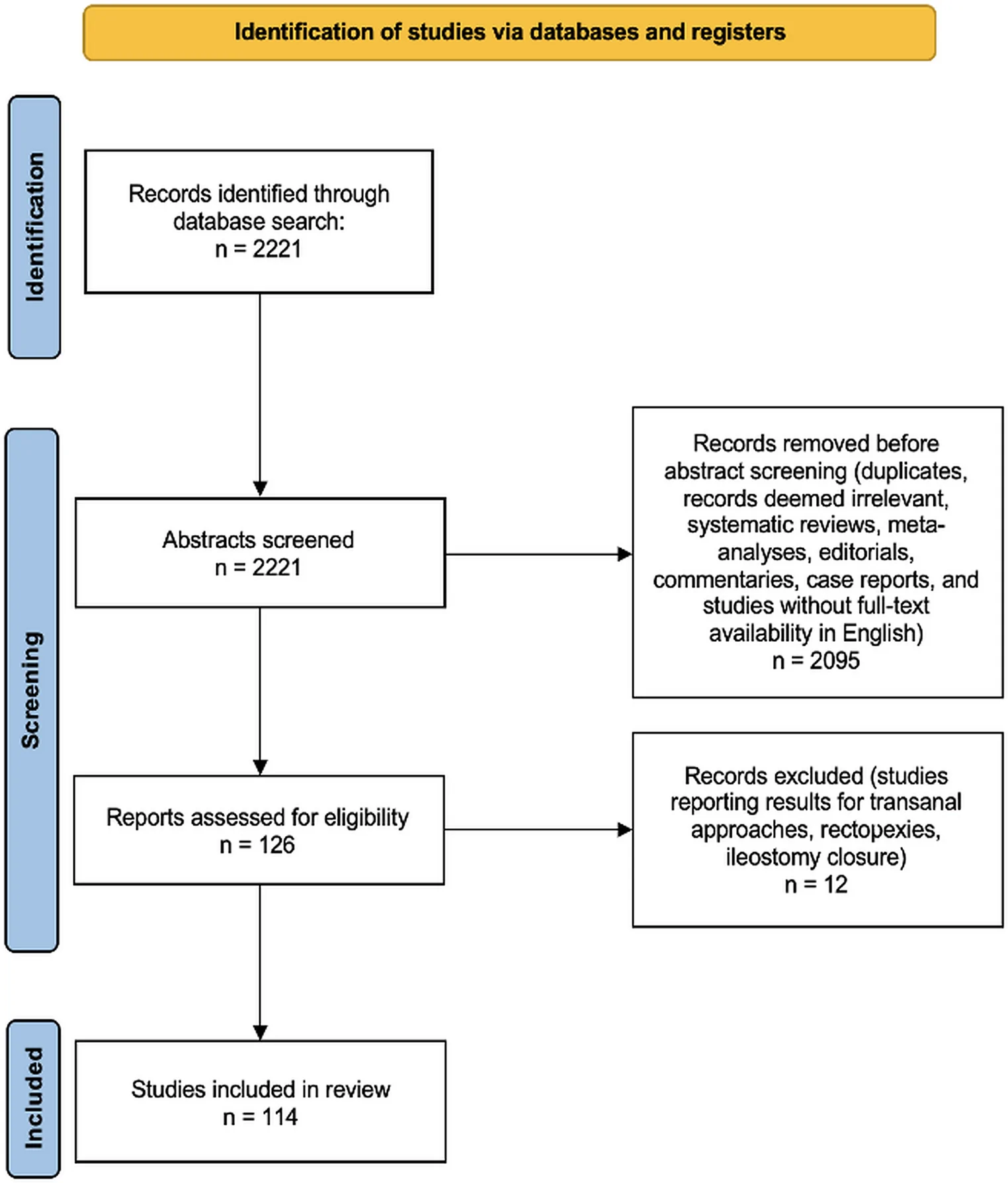
PRISMA flowchart

All titles and abstracts of the identified studies were independently screened by two of the authors (FN and FW). Randomized, prospective, and retrospective clinical studies reporting learning-curve data for colorectal surgery were included. Duplicates and records deemed irrelevant were excluded. In cases where clarification was needed, a consensus was reached through discussion with a senior author of the study (CK). In addition, systematic reviews, meta-analyses, editorials, commentaries, case reports, and studies without full-text availability in English were excluded from the analysis. All parameters used to assess the learning curve were included. The definition of the learning curve was adopted according to the definition of each individual study. Further exclusion criteria were articles not providing a case number or range at which the learning curve was attained, articles that compared preexisting data and articles reporting emergency procedures in colorectal surgery. The study selection procedure is presented in the PRISMA flow chart (Fig. 1).

### Outcome parameters

The primary endpoint was the number of surgical procedures required to overcome or exceed the learning curve as determined by the authors of the studies. Secondary endpoints included the method chosen to describe the learning curve (statistical calculation e.g. CUSUM, or arbitrary, meaning the number of procedures per learning phase was arbitrarily defined), the number of phases described in the learning curve, the number of flexion points, the number of included surgeons, previous laparoscopic and/or robotic surgical experience where reported, and the studies baseline characteristics. Where reported, prior laparoscopic and robotic surgical experience of included surgeons was systematically recorded to contextualize potential skill-transfer effects. Cumulative sum (CUSUM) analysis is a statistical method that graphically tracks cumulative performance over time for either dichotomous or continuous variables^22^. Clinical outcomes during the different learning phases were compared with regard to the following parameters: operative time, console time and docking time in robotic procedures, estimated blood loss (EBL), conversion rate, number of harvested lymph nodes, percentage of postoperative complications (in particular ileus, bleeding, anastomotic leakage, urinary retention) and length of hospital stay (LOS). Data on colon and rectal resections as well as laparoscopic and robotic procedures were analyzed separately.

### Quality assessment

Study quality was evaluated using the Newcastle–Ottawa Scale (NOS) for all included studies. A conservative assessment strategy was applied, focusing on cohort selection, control of confounding, and robustness of outcome assessment, while studies lacking external control groups, multivariable adjustment, or long-term follow-up were rated accordingly. Detailed NOS ratings for all studies in which assessment was feasible are provided in the supplementary material (Table S1).

### Statistical analysis

Reported case numbers required to reach the initial learning-curve threshold were displayed as median and range or interquartile range (IQR) assuming a non-normal distribution due to the low number of studies. Group comparison was performed by the Wilcoxon test. When studies reported more than one threshold for the initial learning phase, the lowest reported threshold was used for the primary analysis. The analyses were stratified by type of resection (colon/rectum) and approach (laparoscopic/robotic) and by the use of statistical methods to assess the learning curve, respectively. All outcome parameters were captured and based on their frequency, the following parameters were further assessed: (1) intraoperative (operative time, blood loss and conversion rate) and (2) postoperative outcomes (overall-, procedure-specific complications and length of hospital stay (LOS)).

An additional analysis was performed to overcome the limitation of heterogeneous learning curve definitions by the study authors. Therefore, the actual number of cases per phase was plotted against the outcome for phase and local polynomial regression models were fitted to the data. Breakpoint estimates were then calculated by segmented relationship assessments separately for rectal and colonic procedures. Based on the breakpoint regression analyses, learning curves were stratified into three predefined learning phases (competency, proficiency, mastery), in accordance with previously published standardized learning-curve frameworks [10, 11]. R version 4.2.3, R Core Team, was used for all analyses and figures.

## Results

### Study selection

The search strategy identified 2,221 records, of which 126 were retained for inclusion following title and abstract screening. Twelve studies were excluded during the full-text screening because they described learning curves for transanal approaches and rectopexies, and one additional study reporting outcomes following ileostomy closure was also excluded. In total, 114 studies were included in the final analysis. Four studies examined, in addition to colon and rectal resections, procedures such as Hartmann’s procedure, Hartmann’s reversal, rectopexy, or stoma formation. In these cases, the number of such interventions was negligible; therefore, the respective studies could be included (Fig. 1).

### Study characteristics

A total of 167 learning curves were described in the 114 studies examined. When classified according to approach, there were 94 laparoscopic, 54 robotic, and 3 open learning curve analyses. Furthermore, 16 studies reported combined approaches (e.g. laparoscopic versus robotic) but analyzed them separately.

Categorized according to the type of surgical intervention, a total of 64 rectal resections and 48 colonic resections were analyzed. The latter were further subdivided into right-sided colectomies and other types of colectomies, such as sigmoid resections or transverse resections. Based on this subdivision, we recorded 32 colectomies and 16 right-sided colectomies. 55 procedures were described as “mixed,” which often included all forms of colorectal surgery.

### Definition of the learning curve

The statistical method most frequently used to assess the learning curve was cumulative sum (CUSUM) analysis, including the risk-adjusted CUSUM (RA-CUSUM), which was applied in a total of 110 analyses (66.4%). In 55 analyses (33.3%), an arbitrary approach was used. Several analyses applied two or more statistical methods to assess the learning curve. Other methods included the ANOVA method, t-test or moving average.

Seventy learning curve analyses identified two phases of the learning curve (57.4%), 35 analyses reported three phases (28.7%), and the remaining analyses described up to ten phases (13%).

In most of the analyses (*n* = 56, 48.7%) a single parameter was used to define the learning curve, 22 analyses (19,1%) used two parameters, and 37 (32.2%) applied three or more parameters.

In the majority of analyses, the learning curve was defined using intraoperative parameters such as operative time (*n* = 133, 79.6%) of analyses. Other outcome parameters comprised conversion rates (*n* = 45, 26.9%) or postoperative complications (*n* = 39, 23.4%), as well as pathological outcomes such as lymph node yield (*n* = 22, 13.2%). In 21 (12.6%) analyses, the composite endpoint “surgical failure” was employed, which was generally defined by a combination of two or more of the following parameters: conversion rate, complication rate, R1 resection, or inadequate lymph node dissection. Table 2 in the supplementary shows further detailed study characteristics.

### Learning curve phases and perioperative outcomes

The median number of procedures required to achieve competence (the first stage of the learning curve) was 31 (IQR 13–50) for colonic resections and 32 (IQR 20–52) for rectal resections irrespective of surgical approach. The detailed results of other intra- and postoperative parameters of all learning curve analyses can be found in Table 1. Continuous improvement across learning curve phases was observed for console time, docking time, and length of stay; for all other parameters, no meaningful trends were identified.

**Table 1:** Intra- and postoperative outcomes in the different phases of the learning curve

Outcome-Parameters	Phase I/Competency	Phase II/Proficiency	Phase III/Mastery
Operative time (min)	228.2(IQR 189.6–306.2)	201.3 (IQR 173.6–264.2)	213.6 (IQR 180.8–264.6)
Console time (min)	152.5 (IQR 100.4–197.0)	136.6 (IQR 100.0–186.6)	120 (IQR 90.1–136.2)
Docking time (min)	6.9 (IQR 6.5–14.0)	6.1 (IQR 5.0–7.3)	6.0 (IQR 5.0–10.0)
Conversion rate (%)	7.0 (IQR 2.2–14.6)	2.6 (IQR 0 -8.1)	3.0 (IQR 0–7.05)
Estimated blood loss (ml)	100 (50–156)	65 (IQR 45–125)	77 (IQR 50–107)
Major complications (%)	8.3 (IQR 3.3–20.0)	7.0 (IQR 3.0–14.0)	7.4 (IQR 2.3–13.0)
Anastomotic leakage (%)	5.0 (IQR 1.3–8.8)	3.2 (IQR 0.0–6.8)	6.7 (IQR 2.3–8,6)
Length of stay	8.0 (IQR 6.4–10.0)	7.7 (IQR 5.6–9.4)	7.0 (IQR 4.5–9.2)

### Comparison of colonic and rectal resections

**Fig. 2:**
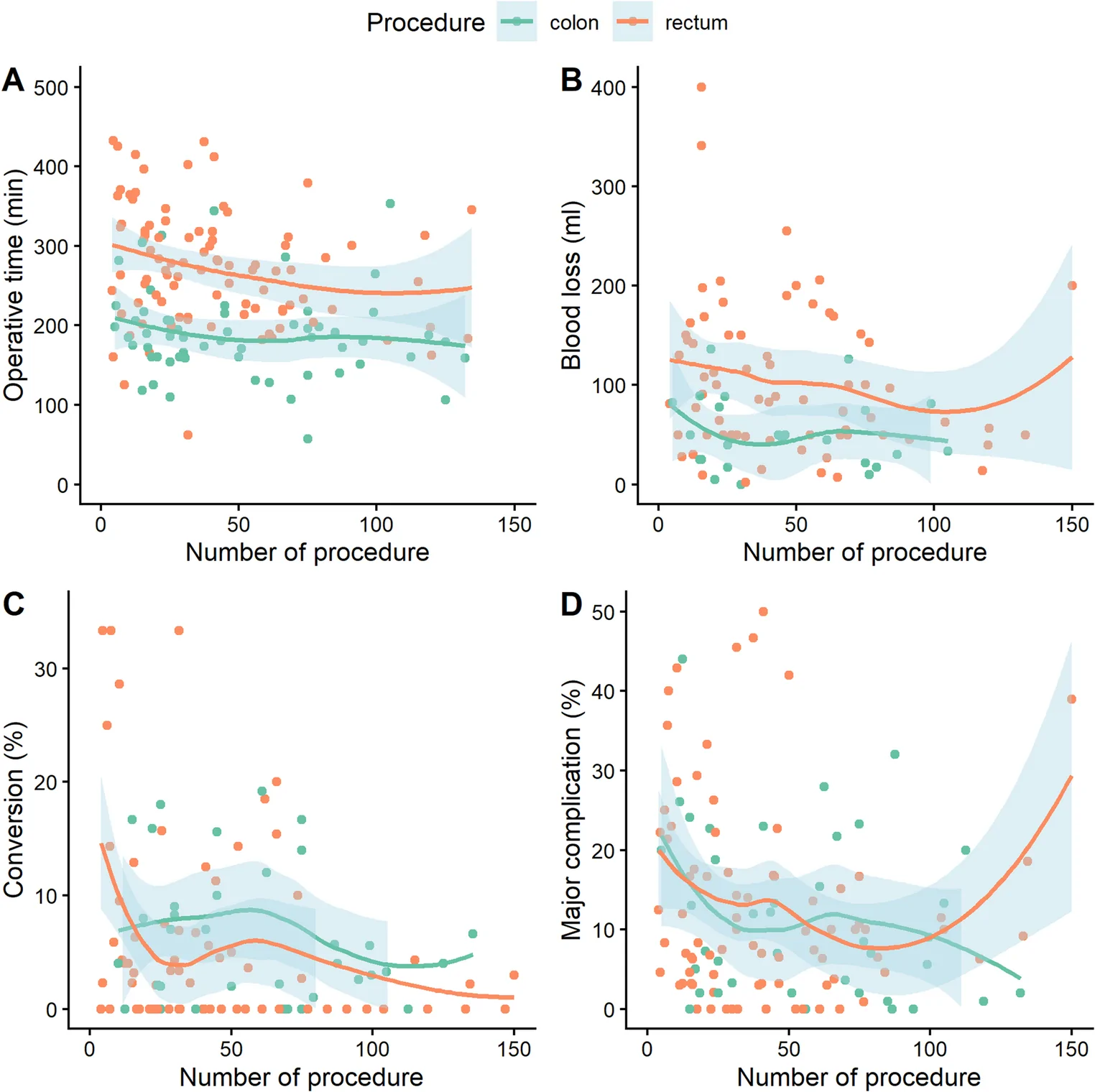
Curved regression models for operative time (**A**), blood loss (**B**), conversion rate (**C**), major complications (**D**) and anastomotic leakage (E) for colonic and rectal resections depending on the number of procedures

Curved regression analyses (Fig. 2) showed progressive improvement in operative time, conversion rate, and major complications with increasing procedural experience, irrespective of learning phase definitions, for both colon and rectal resections independent of surgical approach. These changes appeared more pronounced in rectal resections. Breakpoint analysis identified a change in operative time trends after 38 colonic and 56 rectal procedures, consistent with entry into a plateau phase. Conversion rates declined more substantially after the 75th colonic case and plateaued after the 62nd rectal case. Major complications in colonic resections plateaued after 18 procedures, with a subsequent decline observed after 67 cases. In rectal resections, major complications decreased after 55 procedures and reached a plateau after 82 cases.

### Comparison of laparoscopic and robotic procedures

**Fig. 3:**
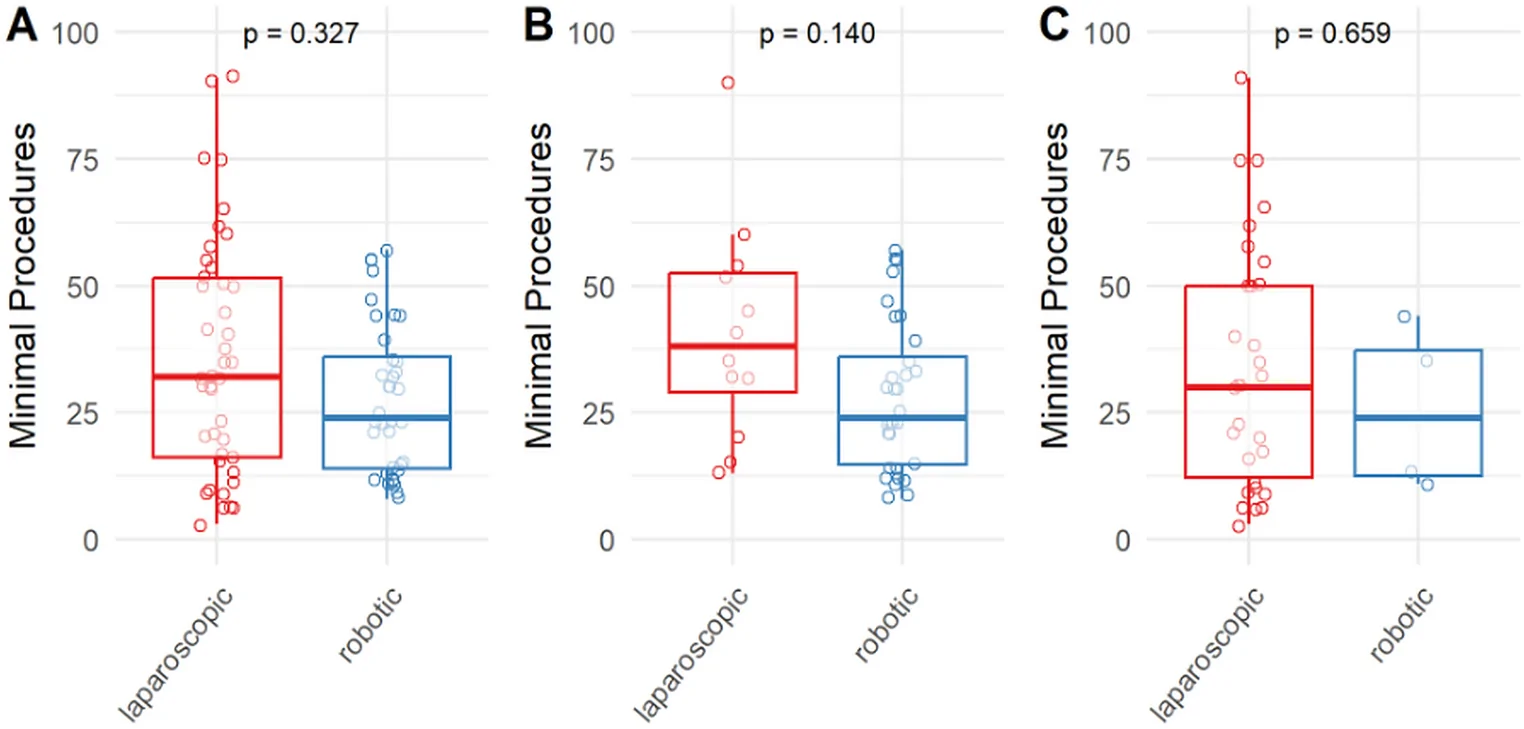
Reported number of procedures required to reach competency in laparoscopic and robotic surgery in all procedures (**A**), rectal resections (**B**) and colonic resections (**C**)

Stratified by surgical approach, reported competency thresholds were numerically lower for robotic than for laparoscopic surgery when applying the learning-curve definitions of the individual studies. For colonic resections, competency was reached after a median of 30 laparoscopic procedures (IQR 14–53) and 24 robotic procedures (IQR 13–37). For rectal resections, the corresponding numbers were 38 laparoscopic procedures (IQR 29–53) and 28 robotic procedures (IQR 15–43) (Fig. 3). These differences were not statistically significant for colonic (*p =* 0.659) or rectal resections (*p =* 0.140). Proficiency thresholds based on intra- and postoperative outcome parameters varied considerably between laparoscopic and robotic resections, with no consistent trend observed based on limited data (Fig. S1).

**Fig. 4:**
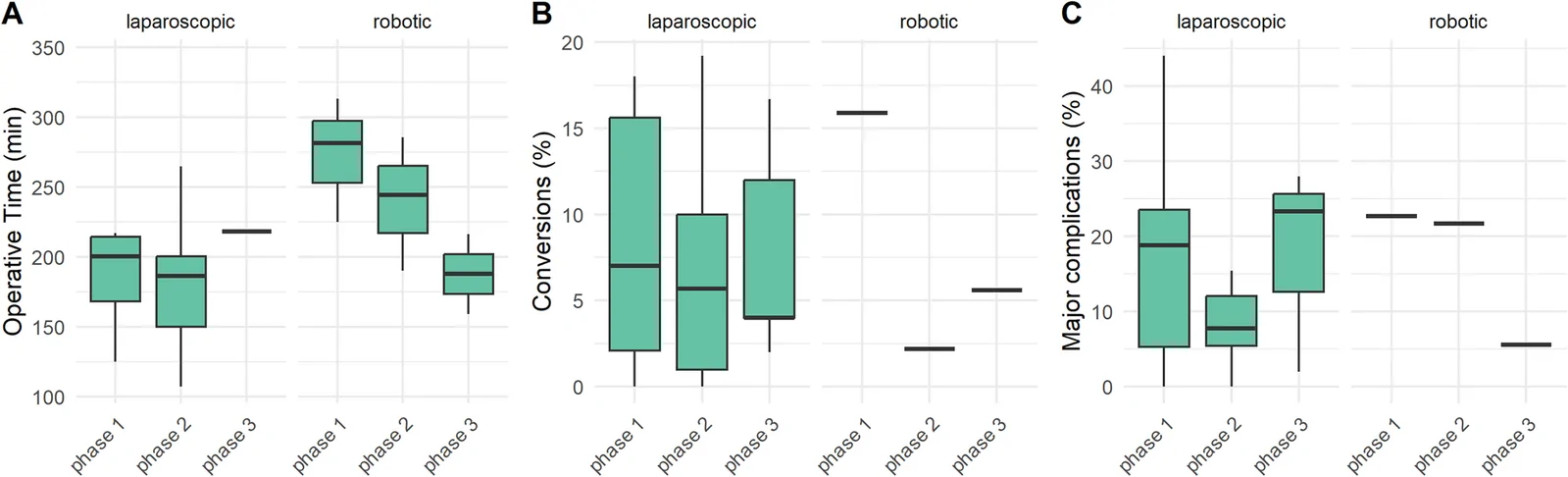
Phases of the learning curves in laparoscopic and robotic colonic resections based on the parameters operative time (**A**), conversion rate (**B**) and major complications (**C**)

**Fig. 5:**
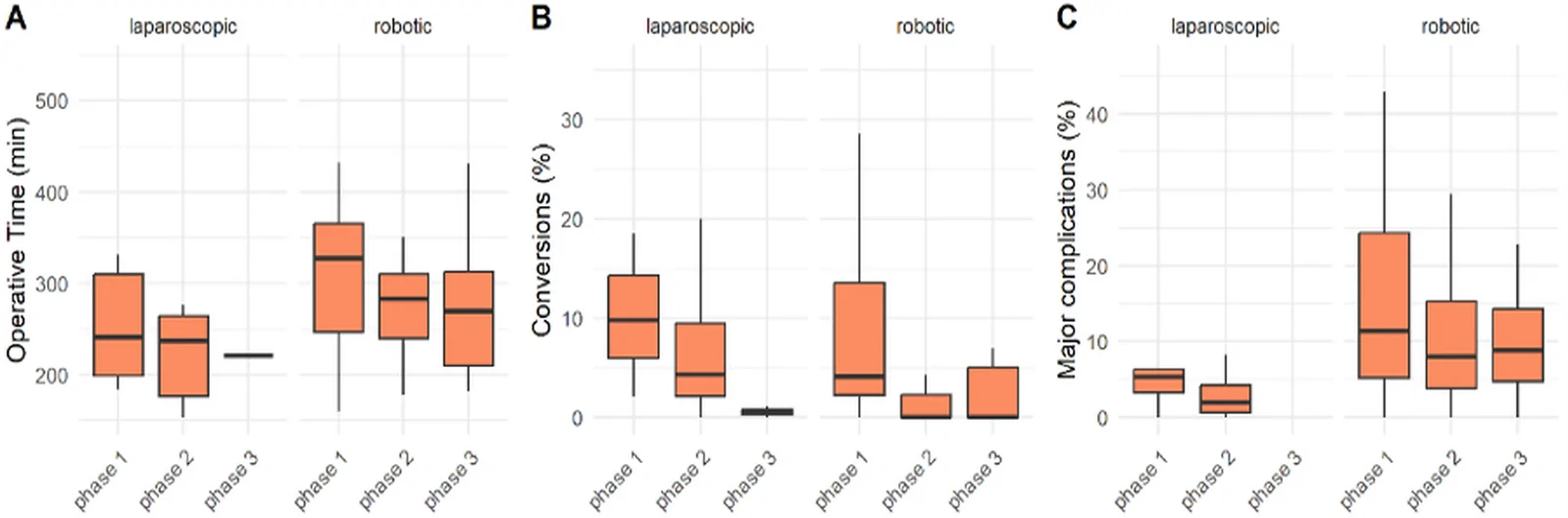
Phases of the learning curves in laparoscopic and robotic rectal resections based on the parameters operative time (**A**), conversion rate (**B**) and major complications (**C**)

Regarding changes across the three learning curve phases based on intra- and postoperative outcomes, numerically greater changes were observed in some outcomes in robotic series, particularly operative time and complication rates (Figs. 4 and 5). However, direct comparison between approaches was limited by data availability. Subgroup breakpoint analyses could only be performed for robotic rectal and laparoscopic colonic resections because these were the only subgroups providing sufficient data for meaningful analysis. Operative time entered the steady phase after 24 rectal surgeries and was stable for colonic procedures. Rectal surgery conversion declined less after 32 procedures and was identical to the overall cohort for laparoscopic colonic operations. There was a continuous reduction in major complications after rectal surgeries and again the identical finding as in the overall cohort in laparoscopic colon surgeries. Table 2 shows the detailed results of intra- and postoperative outcomes of laparoscopic compared to robotic colonic and rectal resections.

**Table 2:** Outcomes regarding operative time, conversion rate and major complications of laparoscopic and robotic colonic and rectal resections in the different phases of the learning curve

Procedure and approach		Colonic resection		Rectal resection	
Parameter and Learning curve phase		Laparoscopic	Robotic	Laparoscopic	Robotic
Operative time (min)	Phase I/ Competency	190.0 (IQR 160.0–209.3)	281.5 (IQR 253.3–297.4)	262.3 (IQR. 199.6–310.8)	327.1 (IQR 236.7–265.9)
	Phase II/ Proficiency	180.4 (IQR 139.3–196.5)	244.3 (IQR 217.2–265.1)	237.4 (IQR 180.8–269.8)	275.2 (IQR 222.6–309.4)
	Phase III/ Mastery	169.5 (IQR 153.3–188.0)	187.7 (IQR 173.4–202.1)	220.6 (IQR 220.3–220.8)	268.0 (IQR 208.5–311.2)
Conversion rate (%)	Phase I/ Competency	7.0 (IQR 2.1–15.6)	15.9 (IQR 15.9–15.9)	9.8 (IQR 6.0–14.3)	4.2 (IQR 2.2–13.6)
	Phase II/ Proficiency	5.7 (IQR 1.0–10.0)	2.2 (IQR 2.2–2.2)	4.3 (IQR 2.1–9.5)	0.0 (IQR 0.0–2.3)
	Phase III/ Mastery	4.0 (IQR 4.0–23.0)	5.6 (IQR 5.6–5.6)	0.6 (IQR 0.3–0.8)	0.0 (IQR 0.0–5.1)
Major complications (%)	Phase I/ Competency	13.0 (IQR 3.3–23.0)	22.7 (IQR 22.7–22.7)	6.4 (IQR 4.8–6.5)	10.3 (IQR 4.3–22.9)
	Phase II/ Proficiency	7.0 (IQR 2.4–11.3)	21.7 (IQR 21.7–21.7)	3.1 (IQR 1.4–7.0)	7.4 (IQR 3.5–15.2)
	Phase III/ Mastery	2.0 (IQR 1.0–23.3)	5.6 (IQR 5.6–5.6)	16.1 (IQR 16.1–16.1)	15.9 (IQR 13.6–17.8)

## Discussion

This systematic review summarizes current evidence on learning curves in minimally invasive colorectal surgery and highlights the wide variation in how learning curves are measured and reported. While most included studies relied on heterogeneous author-defined thresholds, the breakpoint analyses performed in the present study provide data-driven estimates of clinically relevant learning phases. To address this heterogeneity, we propose a standardized reporting framework incorporating a 3-phase model (competency, proficiency, and mastery) describing changes in surgical complexity and perioperative outcomes with increasing experience.

The key findings in this review are as follows: First, increasing procedural experience was consistently associated with improved operative time, conversion rates, and major complications, with more pronounced effects in rectal than in colonic resections. The timing of plateau phases differed between outcomes: operative time stabilized after 56 rectal versus 38 colonic cases, whereas conversion rates plateaued after 62 rectal versus 75 colonic cases. Major complications in colonic resections plateaued after 18 procedures, with a subsequent decline after 67 cases, whereas in rectal resections they decreased after 55 procedures and plateaued after 82 cases. Second, reported competency thresholds were numerically lower in robotic than in laparoscopic series for both colonic (24 vs. 30 cases) and rectal resections (28 vs. 38 cases), although these differences were not statistically significant. Third, some robotic series showed numerically greater changes across learning-curve phases, particularly in operative time and complication rates; however, direct comparison was limited by data availability, as subgroup breakpoint analyses were only feasible for robotic rectal and laparoscopic colonic resections.

These findings highlight that learning curves in minimally invasive colorectal surgery are surgeon-, procedure- and outcome-specific. Rectal resections show greater performance changes with outcome-dependent differences in the timing of plateau phases compared with colonic procedures, reflecting greater technical complexity and possibly pre-existing surgeon experience, as rectal surgery is often performed by surgeons already proficient in colonic resections. Accordingly, learning effects observed in rectal procedures may represent refinement of advanced skills rather than de novo acquisition. Factors such as baseline surgeon experience, institutional volume, and procedural complexity strongly influence how quickly competency is achieved [17–19].

A similar consideration is particularly important when comparing robotic and laparoscopic learning curves. Many early laparoscopic series may reflect the acquisition of minimally invasive surgical skills de novo, whereas surgeons commencing robotic colorectal surgery have commonly already gained substantial laparoscopic experience [6, 8, 9]. Consequently, the numerically lower competency thresholds observed in robotic series may reflect transfer of established minimally invasive skills rather than an inherently shorter learning curve of the robotic platform. Prior laparoscopic experience was inconsistently reported across the included studies and could therefore not be adequately adjusted for. Accordingly, the present data do not allow conclusions regarding the intrinsic learnability of one platform compared with the other. Available evidence suggests that surgeons with substantial laparoscopic experience achieve competency in robotic colorectal surgery more rapidly, while structured training pathways may additionally influence learning and perioperative outcomes [7, 20–22]. Prospective studies evaluating trainees with comparable baseline exposure to laparoscopic and robotic techniques are therefore needed to determine whether platform-specific differences in learning curves exist. Furthermore, the historical timeline introduces a substantial cohort bias: early laparoscopic learning curves evaluated surgical performance during the pioneering phase of minimally invasive colorectal surgery without standardized proctoring or structured simulation. In contrast, recent robotic cohorts benefit from standardized curricula, virtual reality simulation, and optimized perioperative care protocols (e.g., ERAS). Consequently, direct head-to-head comparisons between laparoscopic and robotic learning curves remain inherently limited.

Furthermore, this study showed that while operative time improves early, stabilization of major complication–related outcomes occurs later, suggesting that improvements in operative efficiency may precede stabilization of safety-related outcomes. The fact that different outcomes plateau at different time points underscores the multidimensional nature of surgical learning and supports assessment based on both efficiency- and safety-related outcomes. Operative time is frequently used as a learning-curve endpoint because it reflects efficiency, but its value as a surrogate for clinical outcomes remains limited [23–25]. Only a minority of included studies reported patient-relevant postoperative or oncological outcomes, limiting comparability across learning-curve analyses. Although pooling outcome data helped identify trend changes and plateau phases, standardized outcomes and methods are needed for reliable and comparable assessment in minimally invasive colorectal surgery. Table 3 summarizes several core elements that should be included when designing learning-curve analyses for colorectal surgery.

The learning curves identified in the present study fall within the range reported in the literature. Previous studies suggest competency after 25–35 robotic and 35–60 laparoscopic colorectal procedures, with longer learning curves for rectal than for colonic resections; however, these estimates should be interpreted in light of the differences in baseline surgeon experience, study era, and training environment discussed above. These patterns have been reported across both CUSUM-based and arbitrarily defined analyses [6, 8, 26–28]. At the same time, prior systematic reviews have demonstrated substantial heterogeneity in reported learning-curve thresholds, ranging from fewer than 10 to more than 300 cases, largely driven by the use of single surrogate endpoints and subjective definitions of proficiency. Our findings confirm that learning-curve estimates are highly sensitive to endpoint selection and analytic strategy, reinforcing the notion that surgical learning should be regarded as a multidimensional process rather than a single case number [27, 29]. This pronounced heterogeneity supports the need for a standardized approach to learning-curve assessment.

**Table 3:** Core elements for designing and reporting learning curves in colorectal surgery

Clinically relevant Outcomes [31, 32]	- Intraoperative: operative time, docking time for robotic, bleeding, organ injury, unplanned conversion - Major postoperative complications: anastomotic leaks, Clavien-Dindo grade III or higher - Oncological quality: CRM involvement (≤ 1 mm), lymph node harvest, CME/TME-quality grading (M.E.R.C.U.R.Y. classification) - Patient-centered: return of bowel function, time to oral intake, length of stay, readmission, health-related quality of life
Case Complexity [29, 33]	Miskovic framework: - Tumor characteristics (e.g., rectal height, T-stage) - Patient anatomy (pelvic dimensions, male sex, BMI) - Prior abdominal surgery or adhesions - Inflammatory or bulky disease
Surgeon/Institutional factors [32]	- Proctoring or mentoring availability - Operating room team stability - Institutional case volume - On-call vs. elective environment
Training pathways [7, 23]	- Simulation training (VR or box simulators) - Stepwise curricula (e.g., DaVinci TR100/TR400) - Mentored or proctored surgery - Observation of benchmark procedures - Preclinical skill assessment
Statistical methods/reporting [17, 27, 31, 34]	- CUSUM, risk-adjusted CUSUM or regression-based and breakpoint analyses - Clear defining and stating of start, slope and plateau of the learning curve according the provided 3-phase model - Transparent handling of missing data - Visual reporting using clear graphical formats, including fitted trends or CUSUM plots

Similar to other surgical fields, this systematic review proposes a standardized, case-dependent 3-phase learning model for minimally invasive colorectal surgery, comprising competency, proficiency, and mastery, with structured learning curve reporting that reflects distinct changes in intraoperative and postoperative outcomes as experience increases [10–12, 30]. In the competency phase, surgeons acquire the ability to perform the procedure safely, typically under supervision and in carefully selected low-risk patients, with rapid improvements in intraoperative efficiency and more gradual gains in postoperative outcomes. Reaching competency allows independent performance without direct supervision. The proficiency phase is characterized by increasing consistency and standardization of technique, enabling surgeons to manage more complex cases while complication and conversion rates stabilize and improve. In the mastery phase, surgeons achieve advanced expertise, allowing safe performance of highly complex procedures and effective management of intraoperative challenges, with perioperative and oncological outcomes consistently exceeding benchmark standards. In phase III (mastery), minor non-linear fluctuations in operative time (Table 1) reflect a shift toward expanded surgical indications, wherein surgeons routinely undertake higher-complexity cases (e.g., advanced T-stage, re-operations, elevated BMI, or narrow pelvic anatomy) once technical competence and proficiency have been established. Similarly, the observed increase in major complication rates during Phase III of rectal resections (Table 2) is largely attributable to case selection bias, as surgeons in the mastery phase frequently tackle technically demanding low rectal resections and high-risk patient cohorts. Rather than relying on time-based definitions, this model emphasizes case-based experience as the primary determinant of surgical learning.

### Proposed standards for learning-curve assessment in colorectal surgery

To align future research with the three-phase learning model (competency, proficiency, mastery) and to improve comparability across studies, several core elements should be included when designing and reporting learning-curve analyses for colorectal surgery. These elements are summarized in Table 3.

### Limitations

This review has several limitations. Most included studies were retrospective, with limited reporting of case complexity, surgeon experience, institutional factors, and training structures, precluding adequate adjustment for key confounders. Critical appraisal using the Newcastle–Ottawa Scale (NOS) revealed generally acceptable cohort selection and outcome assessment but limited comparability, as many studies lacked control groups or multivariable analyses, resulting in predominantly moderate methodological quality. Furthermore, substantial heterogeneity in learning-curve definitions, outcome selection, and analytical methods (e.g. CUSUM vs. arbitrary thresholds) limits comparability across studies and hampers the identification of universal benchmarks.

Importantly, laparoscopic and robotic learning curves were derived from different surgical eras and from surgeons with potentially different baseline experience. Differences in prior minimally invasive experience and the evolution of surgical training, including simulation, structured curricula, mentoring, and proctoring, could not be adequately controlled for and may confound comparisons between approaches. Therefore, the observed numerical differences should not be interpreted as evidence that the robotic platform is intrinsically easier or faster to learn.

Because learning-curve data were pooled across heterogeneous studies, the reported thresholds should be interpreted as descriptive estimates rather than universal cut-offs and require consideration of patient-, disease-, surgeon-, and center-specific factors.

## Conclusions

Learning curves in minimally invasive colorectal surgery are heterogeneous and strongly influenced by procedure type, surgeon experience, and training environment. Although robotic series report numerically lower competency thresholds, current evidence does not establish an intrinsically shorter robotic learning curve because prior laparoscopic experience, training pathways, and study era limit direct comparison. Standardized assessment and prospective studies with comparable baseline exposure are required for valid comparison between approaches.

## Supplementary Information

Supplementary Material 1.

## Data Availability

All data analyzed in this study were obtained from previously published studies identified through a systematic literature search. No new datasets were generated for this systematic review. Data extracted and analyzed during the current study are available from the corresponding author upon reasonable request.
