# Supplementary material for: Learning curves in robotic and laparoscopic colorectal surgery: a systematic review
Source: J Robot Surg. 2026 Oct 3;20(1):1050. doi: 10.1007/s11701-026-04024-x (PMC13634005; doi:10.1007/s11701-026-04024-x)
Supplement: Supplementary file 1 [file 11701_2026_4024_MOESM1_ESM.docx]

Supplementary Materials

Table S1: Core elements for designing and reporting learning curves in colorectal surgery.

| Study (First author, year) | Selection  (0–4) | Comparability  (0–2) | Outcome  (0–3) | Total NOS Score  (0–9) |
| --- | --- | --- | --- | --- |
| Choi et al., 2024 | 4 | 0 | 3 | 7/9 |
| Gao et al., 2024 | 4 | 0 | 3 | 7/9 |
| Vela et al., 2024 | 4 | 2 | 3 | 9/9 |
| Saqib et al., 2023 | 3 | 1 | 3 | 7/9 |
| Nann et al., 2023 | 3 | 1 | 3 | 7/9 |
| Zaepfel et al., 2023 | 3 | 2 | 3 | 8/9 |
| Wang et al., 2023 | 3 | 1 | 3 | 7/9 |
| Kolehmainen et al., 2023 | 3 | 1 | 3 | 7/9 |
| Cuk et al., 2023 | 3 | 1 | 3 | 7/9 |
| Burghgraef et al., 2023 | 3 | 2 | 3 | 8/9 |
| Hung et al., 2023 | 3 | 1 | 3 | 7/9 |
| Wong et al., 2023 | 3 | 1 | 3 | 7/9 |
| Rosen et al., 2023 | 3 | 1 | 3 | 7/9 |
| Arquillière et al., 2023 | 3 | 2 | 3 | 8/9 |
| Wong et al., 2022 | 3 | 1 | 3 | 7/9 |
| Lin et al., 2022 | 4 | 1 | 3 | 8/9 |
| Sugishita et al., 2022 | 3 | 1 | 3 | 7/9 |
| Kazi et al., 2022 | 3 | 1 | 3 | 7/9 |
| Giani et al., 2022 | 3 | 1 | 3 | 7/9 |
| Ding et al., 2022 | 3 | 1 | 3 | 7/9 |
| Kim et al., 2022 | 3 | 1 | 3 | 7/9 |
| Sukumar et al., 2022 | 3 | 1 | 3 | 7/9 |
| Perivliotis et al., 2022 | 3 | 1 | 3 | 7/9 |
| Goksoy et al., 2022 | 3 | 1 | 3 | 7/9 |
| Parascandola et al., 2021 | 3 | 1 | 3 | 7/9 |
| Noh et al., 2021 | 3 | 1 | 3 | 7/9 |
| Huerta et al., 2021 | 4 | 1 | 3 | 8/9 |
| Gachabayov et al. 2021 | 3 | 2 | 3 | 8/9 |
| Lee et al., 2021 | 4 | 2 | 3 | 9/9 |
| Zeng et al., 2021 | 4 | 1 | 3 | 8/9 |
| Manigrasso et al., 2021 | 4 | 0 | 2 | 7/9 |
| Nasseri et al., 2021 | 3 | 0 | 3 | 6/9 |
| Gkionis et al., 2020 | 3 | 0 | 3 | 6/9 |
| Yao et al., 2020 | 3 | 0 | 3 | 6/9 |
| Miki et al., 2020 | 4 | 0 | 3 | 7/9 |
| Symer et al., 2019 | 4 | 2 | 3 | 9/9 |
| Lohsiriwat et al., 2019 | 3 | 1 | 3 | 7/9 |
| Blumberg et al., 2019 | 4 | 0 | 3 | 7/9 |
| Raimondi et al., 2018 | 3 | 0 | 3 | 6/9 |
| Ichikawa et al., 2018 | 4 | 0 | 3 | 7/9 |
| Shaw et al., 2018 | 4 | 0 | 3 | 7/9 |
| Guend et al., 2017 | 4 | 0 | 3 | 7/9 |
| Parisi et al., 2017 | 3 | 1 | 3 | 7/9 |
| Odermatt et al., 2017 | 4 | 2 | 3 | 9/9 |
| Weyhe et al., 2017 | 4 | 1 | 3 | 8/9 |
| Ichikawa et al., 2017 | 4 | 1 | 3 | 8/9 |
| Kim, Lee et al., 2016 | 3 | 0 | 3 | 6/9 |
| Tsai et al., 2016 | 3 | 0 | 3 | 6/9 |
| Foo et al., 2016 | 3 | 0 | 3 | 6/9 |
| Kim, Han et al., 2016 | 3 | 0 | 3 | 6/9 |
| Jenkins et al., 2016 | 4 | 0 | 3 | 7/9 |
| Mackenzie et al., 2016 | 4 | 2 | 3 | 9/9 |
| Kim, Kim et al., 2015 | 3 | 1 | 3 | 7/9 |
| Yamaguchi et al., 2015 | 3 | 0 | 3 | 6/9 |
| Melich et al., 2015 | 4 | 0 | 3 | 7/9 |
| Tevlin et al., 2015 | 4 | 0 | 3 | 7/9 |
| Heroor et al., 2015 | 3 | 0 | 3 | 6/9 |
| Trincado et al., 2014 | 3 | 0 | 3 | 6/9 |
| Mackenzie et al., 2014 | 3 | 1 | 3 | 7/9 |
| Luján et al., 2014 | 3 | 0 | 3 | 6/9 |
| Kim, Choi et al., 2014 | 3 | 1 | 3 | 7/9 |
| Park, Kim et al., 2014 (24902812) | 3 | 1 | 3 | 7/9 |
| Park, Kim et al., 2014 (25437022) | 4 | 0 | 3 | 7/9 |
| Melich et al., 2014 | 3 | 0 | 3 | 6/9 |
| Jimenez-Rodriguez et al., 2013 | 3 | 0 | 3 | 6/9 |
| Mackenzie et al., 2013 | 3 | 1 | 3 | 7/9 |
| Chen et al., 2013 | 3 | 0 | 3 | 6/9 |
| Sng et al., 2013 | 3 | 0 | 3 | 6/9 |
| Hopping et al., 2013 | 3 | 0 | 2 | 6/9 |
| Haas et al., 2013 | 3 | 1 | 3 | 7/9 |
| Yamamoto et al., 2013 | 3 | 0 | 3 | 6/9 |
| Pendlimari et al., 2012 | 3 | 1 | 3 | 7/9 |
| Xiao et al., 2012 | - | - | - | -Evaluation not possible |
| Li et al., 2012 | 3 | 0 | 3 | 6/9 |
| Bokhari et al., 2011 | 3 | 0 | 3 | 6/9 |
| Akiyoshi et al., 2011 | 3 | 0 | 3 | 6/9 |
| Kye et al., 2011 | 4 | 0 | 3 | 7/9 |
| Spector et al., 2011 | 3 | 0 | 3 | 6/9 |
| Böttger et al., 2011 | 4 | 0 | 3 | 7/9 |
| Hwang et al., 2010 | 3 | 0 | 3 | 6/9 |
| Ozturk et al., 2010 | 3 | 0 | 2 | 5/9 |
| Waters et al., 2010 | 3 | 1 | 2 | 6/9 |
| Son et al., 2010 | 3 | 2 | 2 | 7/9 |
| Maeda et al., 2010 | 3 | 1 | 2 | 6/9 |
| Park, Choi et al., 2009 (18941844) | 3 | 1 | 2 | 6/9 |
| Park, Choi et al., 2009 (19116741) | 3 | 1 | 2 | 6/9 |
| Choi et al., 2009 | 3 | 1 | 2 | 6/9 |
| Li et al., 2009 | 3 | 1 | 2 | 6/9 |
| Scheer et al., 2009 | 3 | 2 | 2 | 7/9 |
| Fukunaga et al., 2008 | 3 | 1 | 2 | 6/9 |
| Kim et al., 2007 | 3 | 1 | 2 | 6/9 |
| Chen et al., 2007 | 3 | 1 | 2 | 6/9 |
| Charron et al., 2007 | 3 | 1 | 2 | 6/9 |
| Kang et al., 2007 | 3 | 1 | 2 | 6/9 |
| Park et al., 2007 | 4 | 1 | 2 | 7/9 |
| Avital et al., 2006 | 3 | 1 | 2 | 6/9 |
| Tekkis et al., 2005 | 3 | 1 | 2 | 6/9 |
| Jovine et al., 2005 | - | - | - | -Evaluation not possible |
| Shah et al., 2005 | 3 | 0 | 2 | 5/9 |
| Dincler et al., 2003 | 3 | 1 | 2 | 6/9 |
| Caravati et al., 2003 | 3 | 0 | 2 | 5/9 |
| Schlachta et al., 2001 | 3 | 1 | 2 | 6/9 |
| Lauter et al., 2001 | 3 | 0 | 2 | 5/9 |
| Konradt et al., 1998 | 3 | 0 | 2 | 5/9 |
| Lorenz et al., 1998 | - | - | - | - (Evaluation not possible) |
| Agachan et al., 1996 | - | - | - | - (Evaluation not possible) |
| Reissmann et al., 1996 | 3 | 0 | 2 | 5/9 |
| Simons et al., 1995 | 3 | 0 | 2 | 5/9 |
| Wishner et al., 1995 | 3 | 0 | 2 | 5/9 |
| Senagore et al., 1995 | 3 | 0 | 2 | 5/9 |

Table S2: Characteristics of included studies

| References | Year | Surgeon (n) | Approach | Patients (n) | Analysis method | Learning curve phases (n) | Length of learning curve (n) | Factors |
| --- | --- | --- | --- | --- | --- | --- | --- | --- |
| **Colon** | | | | | | | | |
| Senagore AJ | 1995 | - | Laparoscopic | 60 | Arbitrary | 3 | - | OT |
| Simons AJ | 1995 | 4 | Laparoscopic | 144 | Arbitrary | 2 | 6-21 | OT |
| Lauter DM | 2001 | - | Laparoscopic | 150 | Arbitrary | 3 | 50 | OT, conversion, LOS, Comp |
| Dincler S | 2003 | 2 | Laparoscopic | 338 | CUSUM, Moving Average | 2 | 75 | OT, Comp, conversion |
| Tekkis PP | 2005 | - | Laparoscopic | 900 | RA-CUSUM | - | 55-62 | OT, readmission, conversion, Comp |
| Chen W | 2007 | 8 | Laparoscopic | 287 | Arbitrary | 4 | - | OT, conversion, Comp, LOS, readmission |
| Kim J | 2007 | 1 | Laparoscopic | 100 | ANOVA | 2 | 38 | OT |
| Fukunaga Y | 2008 | 1 | Laparoscopic | 404 | Arbitrary | - | 40 | OT, EBL, conversion, Comp |
| Scheer | 2009 | - | Laparoscopic | 487 | ANOVA | - | - | OT |
| Sarli | 2009 | 2 | Laparoscopic | 181 | - | 2 | 91 | - |
| Choi DH | 2009 | 3 | Laparoscopic | 138 | CUSUM, Moving average | 2 | 30 | OT, surgical failure |
| Ozturk E | 2010 | 1 | Laparoscopic | 200 | Arbitrary | 2 | 50 | OT |
| Kye B-H | 2011 | 2 | Laparoscopic | 117 | CUSUM | 2 | 9 | OT, conversion, Comp |
| Akiyoshi T | 2011 | 1 | Laparoscopic | 108 | Moving average | 2 | 35 | OT |
| Yamamoto M | 2013 | 1 | Laparoscopic | 120 | Moving average | 2 | 30 | OT |
| Haas EM | 2013 | 1 | Laparoscopic | 54 | CUSUM, RA-CUSUM | 2 | 30 | OT, surgical failure |
| Hopping JR | 2013 | - | Laparoscopic | 20 | Arbitrary | 2 | 10 | OT |
| Melich G | 2014 | 1 | Laparoscopic | 81 | CUSUM, Moving average | - | - | OT, Comp, surgical failure |
| Kim CW | 2015 | 1 | Laparoscopic | 113 | CUSUM, RA-CUSUM, Moving average | 2 | 65 | OT, surgical failure |
| Mackenzie H | 2016 | 741 | Laparoscopic | 16701 | RA-CUSUM | - | 3 | Conversion, LOS, Reintervention, Mortality |
| Kim CW | 2016 | 1 | Laparoscopic | 64 | CUSUM, Moving average | 2 | 23 | OT, surgical failure |
| Weyhe D | 2017 | 7 | Laparoscopic | 161 | Arbitrary | - | 6 | OT |
| Parisi A | 2017 | 1 | Robotic | 108 | CUSUM, RA-CUSUM | 3 | 44 | Comp, pathological outcomes |
| Raimondi P | 2018 | 1 | Robotic | 23 | CUSUM | 2 | 13 | Console time, anastomosis time |
| Blumberg D | 2019 | 1 | Mixed | 122 | Arbitrary | 2 | - | OT, conversion, LNH, LOS, Comp |
| Miki H | 2020 | 5 | Laparoscopic | 112 | Arbitrary | 2 | 9 | OT |
| Giani A | 2022 | 3 | Mixed | 202 | CUSUM, RA-CUSUM | 2-4 | 17-20 | OT, surgical failure |
| Lin C-Y | 2022 | 2 | Mixed | 74 | CUSUM | 2 | 31 | OT |
| Cuk P | 2023 | - | Laparoscopic | 61 | CUSUM | 2 | 32 | OT |
| Vela J | 2024 | 2 | Laparoscopic | 84 | CUSUM | 3 | 6 | OT |
| Choi MS | 2024 | 1 | Robotic | 39 | CUSUM | 3 | 11 | OT, console time, docking time |
| **Rectum** | | | | | | | | |
| Park IJ | 2009 | 1 | Laparoscopic | 381 | Moving average | 4 | 60 | OT |
| Son G-M | 2010 | 1 | Laparoscopic | 431 | CUSUM, RA-CUSUM, ANOVA, Moving average | 2 | 90 | OT, Comp, conversion, EBL |
| Bokhari MB | 2011 | 1 | Mixed | 50 | CUSUM | 3 | 15 | OT, console time, docking time |
| Sng KK | 2013 | 1 | Robotic | 197 | CUSUM | 3 | 35 | OT, console time, docking time |
| Jimenez-Rodriguez RM | 2013 | 3 | Robotic | 43 | CUSUM | 3 | 11 | OT, surgical failure |
| Park EJ | 2014 | 1 | Mixed | 178 | CUSUM, RA-CUSUM, Moving average | 2 | 41-44 | OT, pathological outcomes |
| Park EJ | 2014 | 1 | Robotic | 130 | CUSUM, RA-CUSUM, Moving average | 3 | 44 | HLN, R1 resections Comp |
| Kim HJ | 2014 | 1 | Robotic | 167 | CUSUM, Moving average | 3 | 32 | OT, conversion, Comp, Resection margin |
| Luján J | 2014 | - | Laparoscopic | 120 | Arbitrary | 2 | - | OT |
| Mackenzie H | 2014 | 1 | Laparoscopic | 636 | CUSUM | 2 | 54 | OT |
| Tevlin R | 2015 | - | Mixed | 62§ | Arbitrary | - | - | OT |
| Melich G | 2015 | 1 | Mixed | 198 | CUSUM, Moving average | 2 | 55 | OT |
| Yamaguchi T | 2015 | 1 | Robotic | 80 | CUSUM | 3 | 25 | OT |
| Foo CC | 2016 | 1 | Robotic | 39 | CUSUM | 3 | 8 | OT, pelvic dissection time |
| Tsai KY | 2016 | 1 | Laparoscopic | 240 | Moving average, ANOVA | 3 | 15 | OT |
| Kim CW | 2016 | 9 | Laparoscopic | 282 | CUSUM, Moving average | - | 13 | OT, surgical failure |
| Odermatt M | 2017 | 2 | Mixed | 384 | CUSUM | - | 15 | OT, conversion, LNH, LOS |
| Mege D | 2018 | 1 | Mixed | 34 | Arbitrary | - | - | - |
| Zeng Z | 2021 | 1 | Mixed | 342 | Arbitrary | 3 | 42 | OT, Comp, Resection margin |
| Lee JM | 2021 | 1 | Robotic | 506 | RA-CUSUM | 2 | 177 | Surgical failure |
| Gachabayov M | 2021 | 4 | Robotic | 221 | CUSUM, RA-CUSUM | - | - | Resection margin, Reoperation |
| Huerta S | 2021 | 5 | Mixed | 273 | Arbitrary | 3 | 90 | OT, EBL |
| Noh GT | 2021 | 5 | Robotic | 269 | CUSUM | 2 | 23-114 | OT, surgical failure, resection margin |
| Parascandola SA | 2021 | 1 | Robotic | 170 | CUSUM | 4 | 55 | OT |
| Sukumar V | 2022 | 2 | Mixed | 120 | CUSUM | 4 | 83 | LNH |
| Kim HJ | 2022 | - | Robotic | 179 | CUSUM | 2-4 | 33-53 | OT, Comp, LNH |
| Ding H-B | 2022 | 1 | Laparoscopic | 108 | CUSUM | 2 | 45 | OT |
| Kazi M | 2022 | 2 | Mixed | 1000 | RA-CUSUM | 10 | 595 | Surgical failure |
| Sugishita T | 2022 | 1 | Robotic | 149 | CUSUM | 3 | 12-32 | OT |
| Arquillière J | 2023 | 9 | Robotic | 270 | CUSUM | 2-4 | 9-21 | OT |
| Rosen SA | 2023 | 1 | Robotic | 83 | Arbitrary | 4 | - | Console time |
| Burghgraef TA | 2023 | 7 | Robotic | 531 | CUSUM | 2 | 12 | Comp, pathological outcomes |
| Kolehmainen CSJ | 2023 | 4 | Robotic | 139 | CUSUM | - | - | Conversion, Comp |
| Wang Y | 2023 | 2 | Mixed | 271 | CUSUM | 2 | 23-32 | OT, surgical failure |
| Zaepfel S | 2023 | 1 | Robotic | 89 | CUSUM | - | 57 | OT |
| Saqib SU | 2023 | 1 | Robotic | 48 | Arbitrary | 4 | 30 | OT, console time |
| Gao Y | 2024 | 5 | Robotic | 89 | CUSUM | 2 | 47 | OT, LNH |
| **Mixed** | | | | | | | | |
| Wishner JD | 1995 | 3 | Laparoscopic | 150 | Arbitrary | 6 | 35 | OT, Comp, conversion, LOS |
| Reissmann P | 1996 | - | Laparoscopic | 100 | Arbitrary | 3 | - | OT, Comp, conversions, LOS |
| Agachen F | 1996 | 4 | Laparoscopic | 167 | Arbitrary | 3 | 50 | OT, Comp, |
| Lorenz EP | 1998 | - | Laparoscopic | 410 | Arbitrary | 2 | 40 | OT |
| Konradt J | 1998 | - | Laparoscopic | 359 | Arbitrary | - | 40 | OT |
| Schlachta CM | 2001 | 3 | Laparoscopic | 461 | Arbitrary | 2 | 30 | OT, Comp, conversion |
| Caravati F | 2003 | 1 | Laparoscopic | 60 | Arbitrary | 2 | 40 | OT |
| Shah PR | 2005 | - | Laparoscopic | 80 | Arbitrary | 3 | - | Conversion |
| Jovine E | 2005 | - | Laparoscopic | 220 | Arbitrary | 3 | - | OT |
| Avital S | 2006 | 1 | Laparoscopic | 100 | Arbitrary | 2 | 50 | OT, Comp |
| Park J-S | 2007 | 1 | Mixed | 197 | Arbitrary | 2 | 37 | OT |
| Kang J-C | 2007 | 1 | Laparoscopic | 60 | ANOVA | 3 | 21 | OT |
| Charron P | 2007 | - | Laparoscopic | - | - | - | 20 | - |
| Li JCM | 2009 | 1 | Laparoscopic | 100 | Arbitrary | - | 45 | OT |
| Park IJ | 2009 | 1 | Laparoscopic | 1014 | CUSUM, Moving average | 9 | 30 | OT |
| Dalton SJ | 2010 | 8 | Laparoscopic | 300 | Arbitrary | - | - | - |
| Abodeely A | 2010 | 1 | Laparoscopic | 48 | Arbitrary | - | - | - |
| Maeda T | 2010 | 1 | Mixed | 5 | CUSUM, Moving average | - | 7-11 | OT, surgical failure |
| Waters JA | 2010 | 1 | Laparoscopic | 100 | Arbitrary | 2 | - | OT |
| Hwang MR | 2010 | 3 | Laparoscopic | 145 | Moving average | 2 | 30 | OT, error rate |
| Böttger TC | 2011 | 1 | Laparoscopic | 180 | Arbitrary | - | - | OT, Comp, LOS |
| Spector R | 2011 | - | Laparoscopic | 100 | Arbitrary | 2 | 8 | OT |
| Li JCM | 2012 | 7 | Laparoscopic | 1031 | CUSUM, Moving average | 2 | 310 | OT, conversion, Comp |
| Xiao Y | 2012 | 1 | Laparoscopic | 189 | Arbitrary | 2 | 15 | OT, EBL, LNH, Comp |
| Pendlimari R | 2012 | 2 | Laparoscopic | 719 | CUSUM | 2 | 105 | OT, Comp |
| Chen G | 2013 | - | Laparoscopic | 100 | Moving average | 2 | 23 | OT |
| Mackenzie H | 2013 | 23 | Laparoscopic | 608 | CUSUM | 2 | 24 | GAS, Comp, conversion, reoperation |
| Trincado MT | 2014 | 6 | Laparoscopic | 313 | CUSUM, Moving average | 2 | 70 | Conversion, LNH |
| Heroor A | 2015 | 1 | Laparoscopic | 101 | Arbitrary | 2 | 20 | - |
| Jenkins JT | 2016 | 8 | Laparoscopic | 750 | CUSUM | - | 32 | DOPS |
| Ichikawa N | 2017 | 5 | Laparoscopic | 42 | CUSUM, Moving average | - | 18 | OT |
| Guend H | 2017 | 5 | Robotic | 349 | CUSUM | 2-3 | 75-78 | OT |
| Shaw DD | 2018 | 2 | Mixed | 110 | Arbitrary | - | 15 | OT, Comp |
| Ichikawa N | 2018 | 5 | Laparoscopic | 42 | CUSUM, Moving average | 2 | 18 | OT, EBL |
| Lohsiriwat V | 2019 | - | Open | 380 | Arbitrary | 5 | 76 | Comp, Readmission |
| Symer MM | 2019 | 3 | Robotic | 2736 | Case sequence analysis | 4 | 27 | Comp |
| Yao H | 2020 | 2 | Robotic | 165 | CUSUM | 2 | 15-42 | OT |
| Gkionis IG | 2020 | 1 | Laparoscopic | 112 | Arbitrary | 2 | 50 | OT |
| Nasseri Y | 2021 | 1 | Robotic | 111 | CUSUM | 3 | 13 | OT, console time |
| Manigrasso M | 2021 | 1 | Robotic | 45 | Arbitrary | 2 | 15 | OT, LNH, conversion, Comp |
| Goksoy B | 2022 | 1 | Laparoscopic | 106 | CUSUM | 3 | 53 | OT |
| Perivoltis K | 2022 | 2 | Laparoscopic | 214 | CUSUM, change-point analysis | 3 | 110 | OT |
| Wong SH | 2022 | 1 | Robotic | 117 | CUSUM | 3 | 44 | OT, console time |
| Wong SW | 2023 | 1 | Robotic | 85 | CUSUM | 2 | 50 | Console time |
| Hung C-Y | 2023 | - | Mixed | 155 | Logistic regression | 5 | - | ERAS compliance, optimal recovery |
| Nann S | 2023 | 4 | Robotic | 193 | Arbitrary | 4 | 12 | OT, docking time |

**Fig. S1** Number of procedures to reach competency in colon and rectal resection in laparoscopic and robotic approach according to estimated blood loss (A), length of hospital stay (B), complications (C), and conversion rate (D)
